# Editorial: Advanced Interpretable Machine Learning Methods for Clinical NGS Big Data of Complex Hereditary Diseases

**DOI:** 10.3389/fgene.2020.600902

**Published:** 2020-10-23

**Authors:** Yudong Cai, Tao Huang, Peilin Jia

**Affiliations:** ^1^School of Life Sciences, Shanghai University, Shanghai, China; ^2^Shanghai Institute of Nutrition and Health, Chinese Academy of Sciences, Shanghai, China; ^3^University of Texas Health Science Center at Houston, Houston, TX, United States

**Keywords:** artificial intelligence, NGS - next generation sequencing, non-invasive prenatal testing (NIPT), WGS - whole-genome sequencing, WES - whole-exome sequencing

Next-generation sequencing (NGS) has revolutionized biomedical research, enabling genome-wide screening of genetic defects. NGS based tests have many applications in Non-Invasive Prenatal Testing (NIPT), early detection of diseases, targeted therapy of various cancers and etiology of rare diseases. There are numerous NGS based genetic test companies and associated data have been accumulated.

As the genomic data increases, it will be a challenge to identify genetic patterns with traditional sampling-based statistical methods. Therefore, advanced machine learning methods, such as deep learning, and Artificial Intelligence (AI) methods can be very beneficial. As an end-to-end method, the deep neural network can extract complex feature patterns automatically and construct prediction models with little manual feature engineering.

Another change the big data has caused is the comeback of instance-based methods or data-driven methods. Unlike the model-based learning or principle-driven methods, the instance-based learning, such as K nearest neighbors, is easy-to-use, easy-to-interpret and has high accuracy when the sample size is big enough to guarantee its performance and the system is too complex to build principle-driven models.

With clinical NGS big data, the genetic causes of various hereditary diseases can be revealed and the shared genetic relationships between diseases can be investigated. Some v very different diseases may share similar genetic causes and should be treated with similar approaches. Some similar diseases may have different genetic causes and should be treated accordingly. The integration of disease network and drug network will become important.

The interpretable model with simple rules is what we need most to transform information exacted from big data to the knowledge that we can master and apply in medical practice. A black box AI algorithm can't appease a worried patient. The interpretable model is not only good for genetic counseling but also essential for knowledge validation and formation. It can also check the correctness of the models and avoid misleading caused by the bias of big data.

The last but not the least change is that in clinical practice, the analysis methods for NGS panel data is quite different from the analysis methods for WGS/WES data which are widely used in the research community. Most research scientists have not faced such challenges and are not even aware of such problems. For clinical panels, we need to re-invent most NGS analysis methods and tools. Such work has mostly been done in industry and hospitals and requires additional research scientist input.

This Research Topic focuses on the challenges of clinical big data analysis in complex genetic diseases, by introducing the latest interpretable machine learning algorithms. There are 22 published articles.

Lv et al. developed a random forest-based sub-Golgi protein classifier rfGPT. The rfGPT used 2-gap dipeptide and split amino acid composition for the feature vectors and was combined with the synthetic minority over-sampling technique (SMOTE) and an analysis of variance (ANOVA) feature selection method. Its accuracy (ACC) was over 90%.

Zhang H. et al. investigated the lung adenocarcinoma (LUAD) and squamous cell lung carcinoma (SCLC) difference on multi-omics scale. With the Boruta method to remove irrelevant features and the MCFS (Monte Carlo Feature Selection) method to identify the significantly important features, they identified 113 key methylation features and 23 key gene expression features.

Wang Y. et al. identified 704 pathogenic genes, 3,848 pathogenic sites, and 2,075 standard phenotypes for underlying molecular perturbations and their phenotypic impact in 3,803 patients with the broad spectrum of intellectual disability (ID). They built the most comprehensive database of an ID phenotyped cohort to date: IDminer http://218.4.234.74:3100/IDminer/, which included the curated ID data and integrated IDpred tool for both clinical and experimental researchers.

Jin et al. studied the biological functions of LINC00356-miR-199a-3p-CDK1/CCNB1 axis in Hepatocellular carcinoma (HCC). Their results proved that LINC00346 could regulate the expression of CDK1/CCNB1 through the competitive adsorption of miR-199a-3p, thereby affecting the p53 signaling pathway and finally regulating the apoptosis, invasion and cell cycle of HCC cells.

Wang H. et al. analyzed the miRNA expression profiles and clinical data of esophageal carcinoma (EC) patients. They found that miR-29c-3p can target CCNA2 to mediate p53 signaling pathway, finally attributing to the inhibition of cell proliferation, migration and invasion, and making cells arrest in G0/G1 phase.

Zhang X. et al. investigated the effects of miR-221-3p in bone marrow mesenchymal stem cell (BMMSC)-derived microvesicles (MVs) on cell cycle, proliferation, and invasion of acute myelocytic leukemia (AML). They discovered that miR-221-3p in BMMSC-derived MVs can regulate AML cell cycle, cell proliferation, and invasion through targeting CDKN1C.

Cheng et al. analyzed the gene expression profiles of 2,343 tumor cells and 1,246 periphery cells. They applied computational methods to screen core biomarkers that can distinguish the discrepancy between Glioblastoma (GBM) tumor and environment (Cheng et al.). Thirty-one important genes were extracted that may be essential biomarkers for GBM tumor cells.

Liu B. et al. collected 10 patients with persistent atrial fibrillation, 10 patients with paroxysmal atrial fibrillation and 10 healthy individuals and did Methylation EPICBead Chip and RNA sequencing. By analyzing the methylation and gene expression data using machine learning-based feature selection method Boruta, they identified the key genes that were strongly associated with AF and found their interconnections.

Hu et al. applied bioinformatics methods for identifying the differentially expressed genes (DEGs) in the lung adenocarcinoma (LUAD) dataset, predicting where the potential target miRNA was expressed and exploring the corresponding downstream target mRNA. They found that exosome-derived miR-486-5p is responsible for cell cycle arrest as well as the inhibition of cell proliferation and metastasis in LUAD via targeting NEK2.

Li et al. proposed a novel method named faster randomized matrix completion for latent disease-lncRNA association prediction (FRMCLDA) by virtue of improved randomized partial SVD (rSVD-BKI) on a heterogeneous bilayer network. Case studies have shown that FRMCLDA is able to effectively predict latent lncRNAs correlated with three widespread malignancies: prostate cancer, colon cancer, and gastric cancer.

Yip et al. developed the Molecular Prognostic Indicators in Cirrhosis (MPIC) database as a representative example of a n omics database tailored for prognostic biomarker validation. MPIC assists cost-effective prognostic biomarker development by facilitating the process of validation and will transform the care of chronic diseases such as cirrhosis. MPIC is freely available at www.mpic-app.org.

Chen et al. presented a novel computational approach to identify potential distinctive features among bacterial subgroups based on a systematic dataset on the gut microbiome from approximately 1,500 human gut bacterial strains. They also established a group of quantitative rules for explaining such distinctions.

Yao et al. analyzed the gene expression profiles of two datasets: one training dataset that includes 144 COPD patients and 194 ILD patients, and one test dataset that includes 75 COPD patients and 61 ILD patients. They identified the 38-gene biomarker and built an SVM (support vector machine) classifier. Its accuracy, sensitivity, and specificity on training dataset evaluated by leave one out cross-validation were 0.905, 0.896, and 0.912, respectively. And on the independent test dataset, the accuracy, sensitivity, and specificity on were as great as and were 0.904, 0.933, and 0.869, respectively.

Xu et al. designed a new model called probability matrix factorization (PMFMDA) for discovering potential disease-related miRNAs. PMFMDA achieved reliable performance in the frameworks of global leave-one-out cross-validation (LOOCV) and 5-fold cross-validation (AUCs are 0.9237 and 0.9187, respectively) in the HMDD (V2.0) dataset, significantly outperforming a few state-of-the-art methods including CMFMDA, IMCMDA, NCPMDA, RLSMDA, and RWRMDA.

Huang et al. proposed an approach based on information entropy and machine learning for computationally identifying histone butyrylation sites. The proposed method achieved 0.92 of area under the receiver operating characteristic (ROC) curve over the training set by 3-fold cross-validation and 0.80 over the testing set by independent test.

Jiang et al. examined the transcriptional changes of Mycobacterium marinum (*M. marinum*), a pathogenic mycobacterial species closely related to *M. tb*, at different stages of resuscitation from hypoxia-induced dormancy. Their study provided valuable insight into the transcriptome changes of *M. marinum* upon resuscitation as well as gene module function of the bacteria during active metabolism and growth.

Zhou et al. enrolled a total of 564 lung adenocarcinoma patients. The relationship between CTTNB1 mutational status and clinicopathologic parameters, the rates of relapse-free survival (RFS) and overall survival (OS), and the mutational status of other genes commonly mutated in lung adenocarcinoma were analyzed. They found that Female patients and non-smokers are likely to harbor CTNNB1 mutation and primary lung adenocarcinoma with mutated CTNNB1 has a poor prognosis.

Wang C. et al. proposed a PU induction matrix completion algorithm based on heterogeneous information fusion (PUIMCHIF) to predict candidate genes involved in the pathogenicity of human diseases. The experimental results of the PUIMCHIF algorithm regarding the three indexes of precision, recall, and mean percentile ranking (MPR) were significantly better than those of other algorithms.

Zhang J. et al. analyzed the gene expression profiles of 156 KRAS mutation samples and other negative samples with two-stage feature selection approach. Forty-one predictive genes for KRAS mutation were identified and a KRAS mutation predictor was constructed. Its leave one out cross-validation MCC was 0.879.

Su et al. built three multivariable Cox models based on prognostic genes selected from the prognostic protein-coding genes (PCGs) and lncRNAs in gastric cancer. The performance of the three models based on features from only PCGs or lncRNAs or from all prognostic genes were systematically compared, which revealed that the features selected from all the prognostic genes showed higher performance than the features selected only from lncRNAs or PCGs.

Liu X. et al. analyzed the circulating tumor-derived DNAs (ctDNAs) fragment length distribution and found that ctDNA fragments were frequently shorter than the normal cell-free DNA (cfDNA). The findings of this study contributed to improving the detection of low-frequency tumor mutations.

Guo et al. conducted a linkage disequilibrium score regression analysis to confirm the strong genetic correlations between asthma, hay fever and eczema and integrated three distinct association analyses (metaCCA multi-trait association analysis, MAGMA genome-wide and MetaXcan transcriptome-wide gene-based tests) to identify shared risk genes based on the large-scale GWAS results in the GeneATLAS database. Their work may provide help on treatment of asthma, hay fever and eczema in clinical applications.

The 22 articles in this Research Topic only covered a small part of the advanced interpretable artificial intelligence applications in clinical NGS and panel data analysis. We hope more and more AI researchers will devote their time and effort into this field, accelerate the clinical applications of AI and eventually help patients.

## Author Contributions

YC, TH, and PJ wrote this editorial. All authors contributed to the article and approved the submitted version.

## Conflict of Interest

The authors declare that the research was conducted in the absence of any commercial or financial relationships that could be construed as a potential conflict of interest.

